# Evolutionary Trends of Perkinsozoa (Alveolata) Characters Based on Observations of Two New Genera of Parasitoids of dinoflagellates, *Dinovorax* gen. nov. and *Snorkelia* gen. nov.

**DOI:** 10.3389/fmicb.2017.01594

**Published:** 2017-08-24

**Authors:** Albert Reñé, Elisabet Alacid, Isabel Ferrera, Esther Garcés

**Affiliations:** Departament de Biologia Marina i Oceanografia, Institut de Ciències del Mar (CSIC) Barcelona, Spain

**Keywords:** apical complex, *Dinophysis*, extrusomes, HAB, life-cycle, parasites, *Parvilucifera*

## Abstract

Parasites are one of the ecologically most relevant groups of marine food webs, but their taxonomic and biological complexity hampers the assessment of their diversity and evolutionary trends. Moreover, the within-host processes that govern parasitoid infection, development and reproduction are often unknown. In this study, we describe a new species of a perkinsozoan endoparasitoid that infects the toxic dinoflagellate *Dinophysis sacculus*, by including observations of its morphology, ultrastructure, life-cycle development and phylogeny. The SSU rDNA sequence and main morphological features were also obtained for a second parasitoid species infecting the bloom-forming dinoflagellate *Levanderina fissa*. Phylogenetic analyses including the sequences obtained show that all known Perkinsozoa species infecting dinoflagellates cluster together. However, sequences of *Parvilucifera prorocentri* and those obtained in this study cluster at the base of the clade, while the rest of *Parvilucifera* representatives form a separated highly-supported cluster. These results, together with differing morphological characters like the formation of a germ-tube, the presence of trichocysts, or the heterochromatin presence in zoospores nucleus justify the erection of *Dinovorax pyriformis* gen. nov. et sp. nov., and *Snorkelia prorocentri* gen. nov. et comb. nov. (=*Parvilucifera prorocentri*). The morphological features and phylogenetic position of these parasitoids represent ancestral characters for the Perkinsozoa phylum, and also for Dinozoa clade, allowing the inference of the evolutionary framework of these Alveolata members.

## Introduction

Recent studies have highlighted the importance of parasitism and their interactions in aquatic environments in determining the structure of planktonic food webs (de Vargas et al., 2015; Lima-Mendez et al., 2015; Cleary and Durbin, 2016). Dinoflagellates (i.e., organisms belonging to the Dinophyceae phylum) are important members of planktonic communities, reaching up to 50% in OTUs (Operational Taxonomic Units) richness and 40% in read abundance based on metabarcoding surveys of protists from the world's surface oceans (Le Bescot et al., 2015). They are affected by a number of different parasitoids, that are organisms that accomplish their life-cycle in an individual and ultimately kill their host, like the chytrid fungi *Dinomyces* Karpov & Guillou, *Parvilucifera* Norén & Moestrup, and *Amoebophrya* Köppen (Jephcott et al., 2016). The two latter belong to the Alveolata lineage. Main groups composing Alveolata are ciliates and a clade named Myzozoa. The latter encompasses among others, the parasitic group of Apicomplexans and the Dinozoa clade, which includes Perkinsozoa and Dinoflagellata. Dinoflagellata includes the Dinophyceae (dinoflagellates) phylum, and other exclusively parasitic groups like the Syndiniales, or Ellobiopsidae.

The Perkinsozoa have been detected in marine and freshwater environments (Bråte et al., 2010; Mangot et al., 2011), sediments (Chambouvet et al., 2014), and abyssal sea floor (Scheckenbach et al., 2010), but the group is mainly represented by environmental sequences so far. The known Perkinsozoa members are organisms that develop endoparasitic trophonts, divide and form a sporangium with biflagellated zoospores, characterized by having an apical complex with an incomplete conoid (Norén et al., 1999). The only Perkinsozoa genera described to date are *Perkinsus* Levine, which includes up to 8 species that infect molluscs like oysters, clams and scallops (Andrews, 1988; Azevedo, 1989; Blackbourn et al., 1998; Casas et al., 2004), *Rastrimonas subtilis* (=*Cryptomonas subtilis* Brugerolle), which infects cryptophytes, although its phylogenetic position remains to be confirmed (Brugerolle, 2002), and the genus *Parvilucifera*, that encompasses dinoflagellates parasitoids. There are 5 species described so far, namely *P. infectans* Norén and Moestrup (Norén et al., 1999), *P. sinerae* Figueroa, Garcés, Massana & Camp (Figueroa et al., 2008), *P. prorocentri* Leander and Hoppenrath (Leander and Hoppenrath, 2008), *P. rostrata* Karpov and Guillou (Lepelletier et al., 2014), and *P. corolla* Reñé, Alacid, Figueroa, Rodríguez, and Garcés (Reñé et al., 2017).

The different species of *Parvilucifera* share most morphological characteristics, and are mainly distinguished by the zoospores disposition in the sporangium during maturation, with subsequent different sporangium appearance, and zoospores shape and characteristics, like the presence of a refractile body (Reñé et al., 2017). They also share the same life-cycle development. Briefly, a free-living flagellated zoospore penetrates a host, transforms into a feeding stage, the trophont, and consumes the cellular content. Then, schizogony takes place and the nucleus begins to divide by mitosis, transforming the trophont into a sporocyte. Subsequently, cytokinesis occurs, producing hundreds of new zoospores to be released into the environment. The whole life-cycle can be completed in about 3–5 days.

In this study, we provide morphological observations, ultrastructural details of different life-cycle stages, and phylogenetic information of a new species of Perkinsozoa parasitoid infecting dinoflagellates, as well as phylogenetic information and general morphological features of a second one. The comparison and differences observed for some morphological characters and their phylogenetic position reveal the need to erect two new genera for *Dinovorax pyriformis* gen. nov. et sp. nov., and *Snorkelia prorocentri* gen. nov et comb. nov. (=*Parvilucifera prorocentri*). This study represents a step beyond to the knowledge of Perkinsozoa diversity, and given that Perkinsozoa occupy a basal phylogenetic position in Dinozoa and Myzozoa, the morphological features observed allow the inference of the evolutionary trends within Perkinsozoa and among Alveolata groups.

## Materials and methods

### Isolation and culture

Seawater samples were obtained using a 10 μm-mesh net on March 2015 and January 2016 in El Masnou Harbor (41°28′39″N; 2°18′47″E), and on January 2017 in Ginesta Harbor (41°15′38″N; 1°55′41″E) and Arenys de Mar Harbor (41°34′45″N; 2°33′20″E), Catalan Coast, NW Mediterranean Sea. Additionally, a sample from La Fosca beach shoreline (41°51′16″N; 3°08′36″E) containing both water and sediment was taken in August 2016. A small volume (~5 mL) of sample was transferred to well-plates and placed in a culture chamber (temperature 20°C, 12:12 light:dark cycle, photon irradiance of 100 μmol photons m^−2^ s^−1^). After 2–3 days, cells of *Dinophysis sacculus* Stein and *Levanderina fissa* (Levander) Moestrup, Hakanen, Hansen, Daugbjerg & Ellegaard were observed being infected by parasitoids. Infected cells were manually isolated and transferred to new well-plates adding host cultured-cells. Because cultures of *Dinophysis* were not available, a set of different dinoflagellate hosts were added to the different wells in order to propagate the infections. Samples with the dinoflagellate *Prorocentrum micans* Ehrenberg were infected and the parasitoid could be successfully cultured. The parasitoid cultures were maintained transferring some infected cells into new wells filled with healthy host approximately once a week. Established strains are listed in Table S1.

### Optical microscopy

Life-cycle development and the morphology of the different stages of *D. pyriformis* were studied transferring the cultured parasitoid species to settling chambers at different moments of infection development. To study nuclear development and divisions, 5 mL of culture containing different life-cycle stages were fixed with 4% formaldehyde and stained with DAPI (4′, 6-diamidine-2′-phenylindole dihydrochloride) at a final concentration of 10 μL mL^−1^. Settling chambers were observed under a phase-contrast Leica DM-IRB inverted microscope (Leica Microsystems, Wetzlar, Germany) equipped with an epifluorescence UV filter set, and connected to a ProgRes C10 (JENOPTIK Laser, Optik, Systeme GmbH, Jena, Germany) digital camera. Cellular measurements were conducted using ProgRes CapturePro software. A time-lapse video was recorded using a Zeiss AxioObserverZ1 inverted microscope equipped with software Zeiss ZEN 2012, taking images of a *P. micans* cell infected by *D. pyriformis* every 2 h for 2 days. Videos were recorded under a Leica-Leitz DM-Il inverted equipped with a Sony NEX-3 camera (Sony, Tokyo, Japan) during zoospores release in order to measure sporangia size and count the number of zoospores present in each sporangium. The biovolume of the sporangium was calculated assuming a spherical shape and not taking into account the volume of the germ-tube, which is empty during zoospores formation. Infections of *Snorkelia* sp. (from La Fosca beach) could not be propagated successfully in culture and it was lost before a complete morphological characterization was accomplished.

### Electron microscopy

Two samples from *D. pyriformis* strain Masnou 2015 (Table S1) were processed for scanning electron microscopy (SEM), one containing free zoospores and another one containing mature sporangia. When free-living zoospores were observed to emerge from sporangia, 2 mL of culture were filtered through a 5-μm filter. The passing-through fraction was collected in a 2-mL Eppendorf tube, fixed with 2% glutaraldehyde (final concentration) and stored at 4°C until processed. Two mL of the culture were also collected when mature sporangia were observed. The sample was directly fixed with 2% glutaraldehyde and stored at 4°C until processed. Later on, osmium tetroxide was added at a final concentration of 2% to the sample already containing the glutaraldehyde and stored again at 4°C for 1 h. Samples were filtered by gravity through 0.8 and 3.0 μm pore size polycarbonate filters respectively, and then washed in seawater for 15 min and in distilled water for 15 min. A subsequent dehydration was carried out in a 25, 50, 75, 90, 96, and 100% ethanol series for ca. 10 min. The final step of 100% ethanol was repeated twice. The filters were critical-point dried and mounted on stubs, sputter-coated with gold-paladium and examined under a HITACHI S-3500N scanning electron microscope (Hitachi High Technologies Corp., Japan) at the Servei de Microscopia Electrònica (ICM-CSIC). Measurements were conducted using ImageJ software. Two mL of culture collected at successive times along the infection process were fixed with 2% glutaraldehyde to observe the ultrastructure of the different life-cycle stages of *D. pyriformis* strain Masnou 2015 under transmission electron microscopy (TEM). Samples treatment and observations were performed at the Centres Científics i Tecnològics de la Universitat de Barcelona (CCiTUB) following the procedures detailed in Reñé et al. (2017).

### PCR amplification, sequencing and phylogenetic analysis

Some mature sporangia of cultures were isolated, transferred to successive drops of seawater and placed into 200 μl PCR tubes. PCR tubes were subjected to three rounds of freeze-thaw, and finally stored at −80°C until processed. SSU rDNA amplification was conducted following the procedures detailed in Reñé et al. (2017). For LSU rDNA region, we used the primer pair D1R (Scholin et al., 1994) and D3B (Hansen et al., 2000) using the same PCR mixture, and PCR conditions as follows: initial denaturation for 5 min at 95°C, 40 cycles of 20 s at 95°C, 30 s at 55°C, and 1 min at 72°C, followed by a final extension step for 7 min at 72°C. LSU sequences of *P. corolla* were also obtained for isolated sporangia from the culture available (strain Estartit). Purification and sequencing were carried out by an external service (Genoscreen, France). Sequencing was done using forward and reverse primers for both primer pairs and a 3730XL DNA sequencer and partial sequences obtained were merged. The obtained sequences were aligned with a selection of environmental sequences covering the diversity of Perkinsozoa, as well as some representatives of other Alveolata groups, obtained from GenBank using the MAFFT v.6 program (Katoh et al., 2002) under –auto option. The alignment was manually checked with BioEdit v. 7.0.5 (Hall, 1999), obtaining 1940 positions for SSU sequences and 1269 positions for LSU sequences. Subsequently, the alignments were trimmed using Gblocks using the less stringent options (Castresana, 2000), resulting in a final alignment of 1611 positions for SSU sequences and 743 for LSU sequences. Phylogenetic relationships were determined as described in Reñé et al. (2017). All sequences obtained were deposited in GenBank under the Accession Numbers MF197549-MF197552 for SSU rDNA and MF197553-MF197556 for LSU rDNA sequences (Table S1).

## Results

### Formal description

Alveolata Cavalier-Smith, 1991

Myzozoa Cavalier-Smith and Chao, 2004

Perkinsozoa Norén and Moestrup, 1999

Parviluciferaceae Reñé and Alacid fam. nov.

*Dinovorax* Alacid and Reñé gen. nov.

DESCRIPTION: Endoparasitoid of dinoflagellates. Sigmoid shaped zoospores penetrate a healthy host and transform into the feeding stage (trophont). Zoospores are biflagellated, with both flagella equal in size but heteromorphic. Both emerge from the anterior half of the cell. When the host is consumed, the trophont forms a germ-tube and transforms into a sporocyte. Numerous new zoospores are then produced and released through the germ-tube once completely formed.

TYPE SPECIES: *Dinovorax pyriformis* Alacid et Reñé sp. nov.

ETYMOLOGY: *Dino*- referring to dinoflagellates, -*vorax* from Latin meaning voracious.

*Dinovorax pyriformis* Alacid et Reñé sp. nov.

DESCRIPTION: Endoparasitoid of dinoflagellates. If forms a germ-tube from where zoospores are released. Zoospores are sigmoid, with two heteromorphic flagella of similar length. The nucleus presents ovoid heterochromatin structures along its periphery. Zoospores possess bipartite trichocysts, a mitochondrion, and lipid droplets. Apical complex is present, with pseudo-conoid, micronemes and rhoptries.

HOLOTYPE: SEM stubs containing free-living zoospores and mature sporangia and resin-embedded samples of the type locality used for TEM containing all life-cycle stages of the parasitoid and have been deposited in the Electronic Microscopy Laboratory of the Institut de Ciències del Mar (ICM-CSIC) from Barcelona, under the code ICM–SEM–2016_AR_15 to 16 and ICM–TEM–2016_AR_17 to 24 respectively.

TYPE LOCALITY: El Masnou harbor, Catalonia, NW Mediterranean Sea (41°28′39″N; 2°18′47″E).

TYPE HOST: *Dinophysis sacculus*

ETYMOLOGY: *pyriformis*, from Latin *pyrum* (pear) and *forma* (shape), referring to the pear-shape of the mature sporangium.

*Snorkelia* Reñé et Alacid gen. nov.

DESCRIPTION: Endoparasitoid of dinoflagellates. It forms a germ-tube from where zoospores are released. Zoospores are reniform. The nucleus has condensed chromatin beneath the nuclear envelope. A refractile body and bipartite trichocysts are present. They possess two heteromorphic flagella, a short posterior flagellum short and a haired anterior one. Apical complex is present, with pseudo-conoid, micronemes and rhoptries.

TYPE SPECIES: *Snorkelia prorocentri* (Leander et Hoppenrath) Reñé et Alacid comb. nov.

ETYMOLOGY: from snorkel (hollow tube used for breathing underwater), of German origin, due to the resemblance of the mature sporangium with Snorks cartoons, which were small beings inhabiting undersea with snorkels on their heads.

*Snorkelia prorocentri* (Leander et Hoppenrath) Reñé et Alacid comb. nov.

BASIONYM: *Parvilucifera prorocentri* Leander et Hoppenrath

### Occurrence of infections

On March 2015, observations of net-samples obtained in El Masnou Harbor resulted in the detection of *Dinophysis* cells infected by parasitoids with general features not corresponding to any known species. *Dinophysis sacculus* and *D. acuminata* were present at abundances of 10^3^ and 10^4^ cell L^−1^ respectively, and *P. micans* was also present at <10^3^ cell L^−1^. On January 2016, moderate abundances of those species were reported again in the same sampling site. Net-samples were collected. After 3-4 days, it resulted in the recurrent observation of infected cells of *Dinophysis*. On January 2017, infected cells were detected in samples obtained from Ginesta and Arenys harbors containing both *D. sacculus* and *P. micans* at abundances <10^3^ cell L^−1^. In all cases, the parasitoid was fed in culture using the dinoflagellate *P. micans* as host. Additionally, on August 2016, a sample was obtained from La Fosca beach with presence of the dinoflagellates *L. fissa* and *Alexandrium taylori* Balech at abundances of <10^4^ cell L^−1^. *L. fissa* cells were observed to be infected by a morphologically similar parasitoid.

### Morphology of *Dinovorax pyriformis* life-cycle stages and infection development

**Figure 1:**
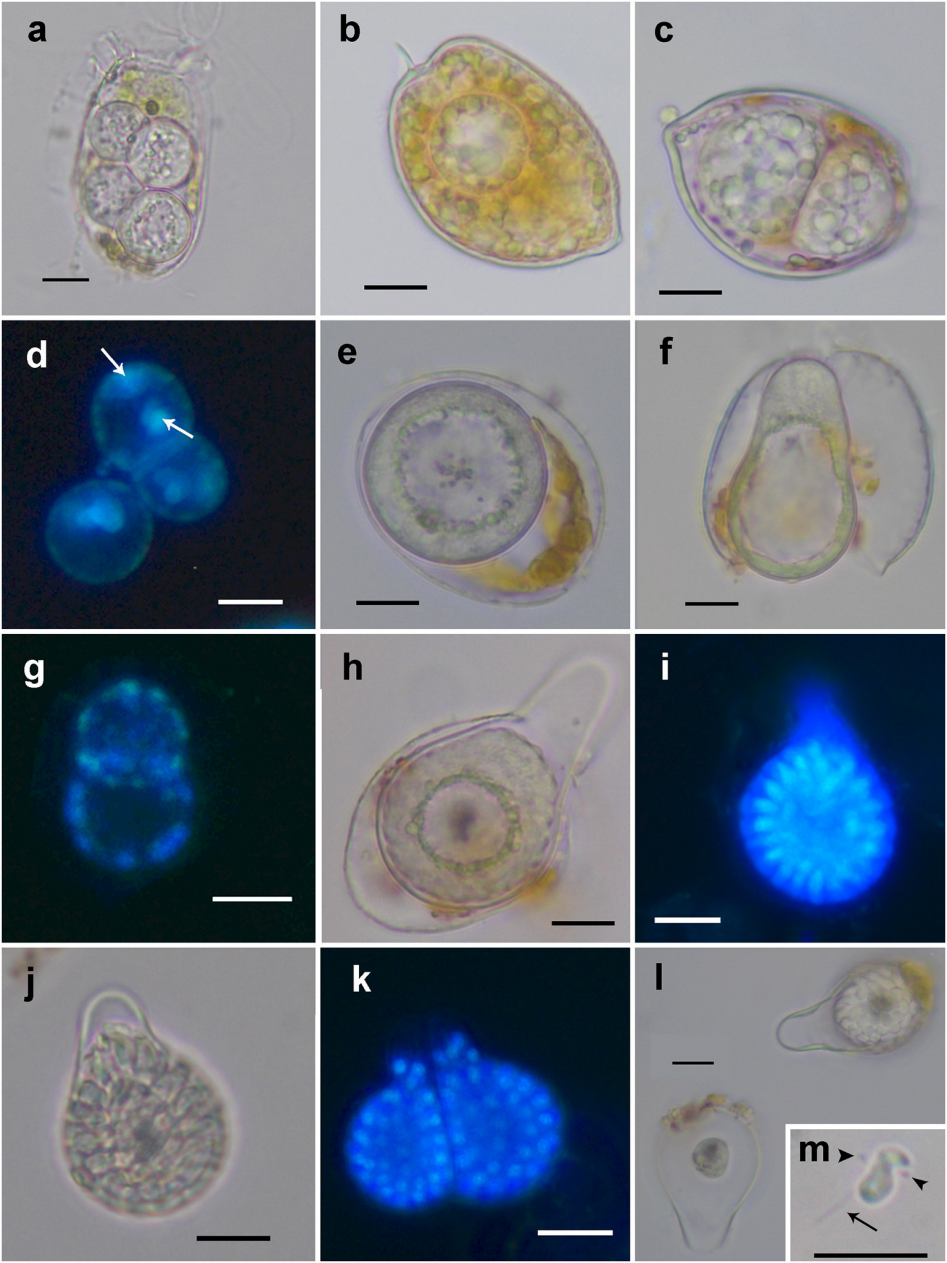
Light microscopy and epifluorescence micrographs showing the life-cycle stages of *Dinovorax pyriformis* during host infection. **(a)** Four early trophonts infecting a *Dinophysis sacculus* cell. **(b)** Early trophont infecting a *Prorocentrum micans* cell. **(c)** Double infection of two late trophonts in a *P. micans* cell. **(d)** Epifluorescence image of three early sporocytes stained with DAPI showing two nuclei (arrows). **(e)** Early sporocyte. The entire cytoplasmatic content aggregates in the periphery while the inner area is occupied by amorphous hyaline material. **(f)** Early sporocyte forming the germ-tube. **(g)** Epifluorescence image of two early sporocytes stained with DAPI showing several nuclei distributed along the cell periphery. **(h)** Early sporocyte after the formation of the germ-tube. **(i)** Epifluorescence image of a late sporocyte stained with DAPI showing numerous elongated nuclei of zoospores in formation arranged around a central body. **(j)** Late sporocyte with zoospores in formation arranged around a central body. **(k)** Epifluorescence image of two mature sporangia with many visible nuclei of mature zoospores. At this late stage, zoospores nucleus is rounded. **(l)** Empty sporangium (left) with only the central body remaining in it and early sporocyte (right). **(m)** Zoospore showing its characteristic sigmoid shape. The anterior (arrowheads) and posterior flagellum (arrow) can be seen. Scale bars = 10 μm.

**Figure 2:**
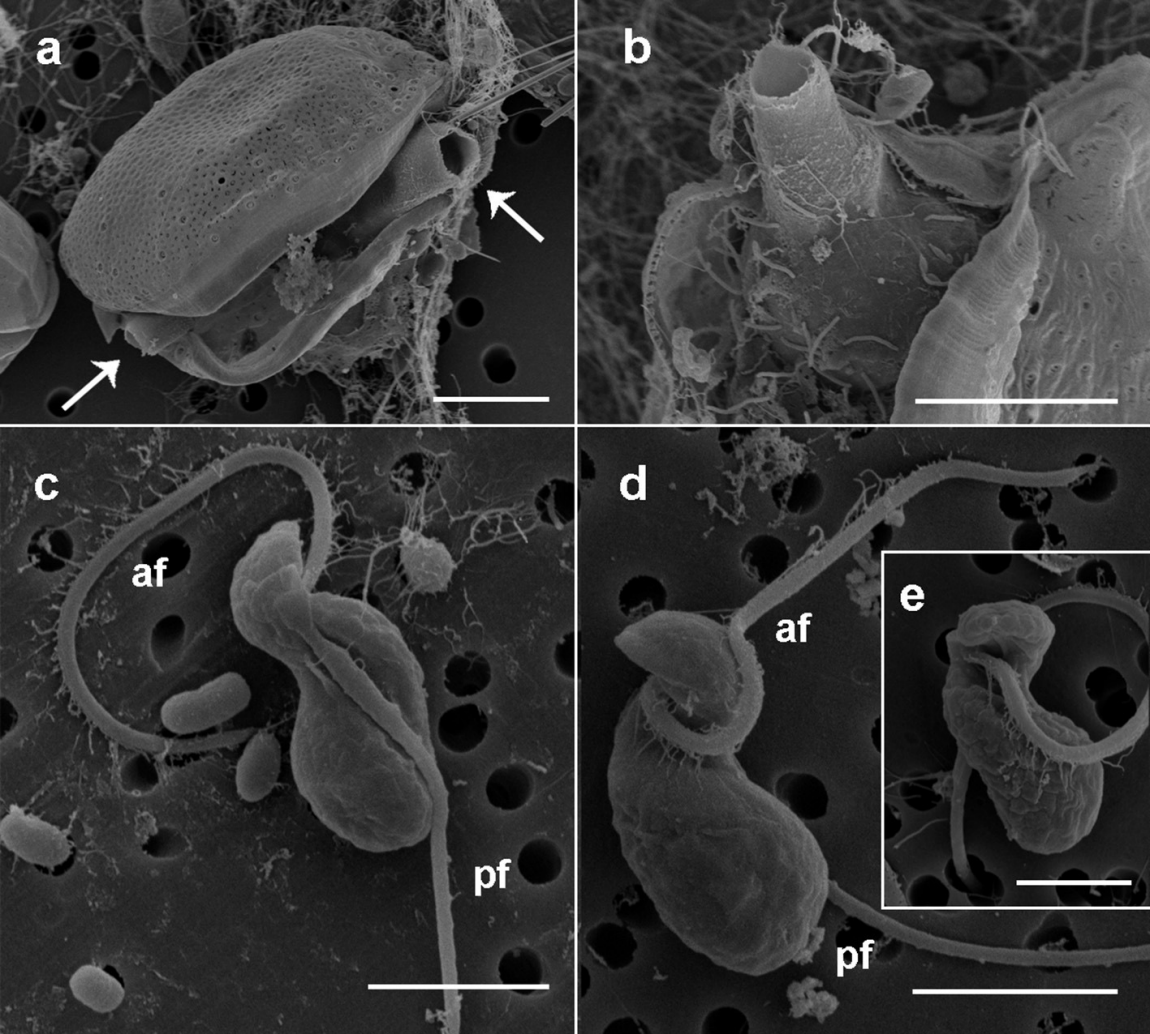
Scanning electron microscopy micrographs of the sporangia and zoospores of *Dinovorax pyriformis*. **(a)** *Prorocentrum micans* cell broken because of the emergence through the sagittal suture of two open germ-tubes (arrows) of *D. pyriformis*. **(b)** Detail of the sporangium inside the host theca and the opened germ-tube emerging out of it. **(c)** Right lateral view of a zoospore. The naked posterior flagellum lies in a cavity running all along the lateral side of the cell. **(d)** Left lateral view of a zoospore. The hairy anterior flagellum encircles the cell to its left side. **(e)** Ventral view of a zoospore showing the insertion point of the anterior flagellum. Scale bars: **(a,b)** = 10 μm, **(c,d)** = 3 μm, **(e)** = 1 μm. af, Anterior flagellum; pf, posterior flagellum.

The detailed observation of infection development in *Prorocentrum* host cultures under light microscopy (LM) allowed to characterize the life-cycle of *D. pyriformis*. The infection occurs when a free-living zoospore penetrates into the dinoflagellate host. Then, it transforms into a spherical trophont and begins to degrade the host cytoplasm content (Figures 1a–c). Up to 4–6 infections can simultaneously occur and develop at the same cell when infecting *D. sacculus* (Figures 1a), whereas only single or double infections were observed on *P. micans* (Figures 1b,c). The trophonts appear vacuolated and gradually grow in size until they occupy the entire host cytoplasm (Figure 1c). Once the host is consumed, the nucleus of the trophont successively divides, and becomes a sporocyte stage (Figure 1d). At this stage, the cytoplasmic content of the parasitoid is located at the periphery, while its central area is occupied by hyaline material (Figure 1e). The germ-tube, also defined as a discharge tube, then develops (Figure 1f), and nuclear divisions continue, resulting in the observation of several smaller nuclei at the cell periphery (Figure 1g). Once the germ-tube is formed, the cytoplasm of the sporocyte retracts, leaving the tube empty during successive stages (Figure 1h). Subsequently, the differentiation of new zoospores begins, showing a radial disposition around a central body (Figures 1h–j). The mature zoospores show a rounded small nucleus (Figure 1k). The sporangium is pear-shaped, with a short germ-tube broad at its base (Figures 1j–l). The germ-tubes emerge through the perivalvar suture of the host *P. micans* (Figures 2a,b) and when zoospores are released, a round residual body sometimes remains in the sporangium of *D. pyriformis* (Figure 1l). The time-lapsed life-cycle development of *D. pyriformis* can be seen in Video S1. Zoospores are 6 μm long and 3 μm wide (*n* = 11) and show a characteristic sigmoid shape and possess two heteromorphic flagella that can be observed under light microscopy (Figure 1m). They have a sigmoid shape in lateral view and the anterior area presents a rostrum (Figures 2c,d). A hairy anterior flagellum emerges ventrally in the cavity formed by the rostrum and encircles it (Figures 2d,e). The posterior flagellum is inserted laterally, positioned into a depression, and runs longitudinally to the posterior end of the cell (Figure 2c). The posterior flagellum shrink at its distal end (not shown). Both flagella are inserted forming a 90° (Figures 2c–e) and are of almost equal length. Zoospores swim slow and smoothly in one direction. Then, they make small jumps to turn direction (Video S2). Sporangia on *P. micans* had a volume of around 3,800–5,500 μm^3^ and were observed to contain between 50 and 80 zoospores respectively, with a ratio of 10–16 zoospores/10^3^ μm^3^ (*n* = 4).

### Ultrastructure of *Dinovorax pyriformis* life-cycle stages

**Figure 3:**
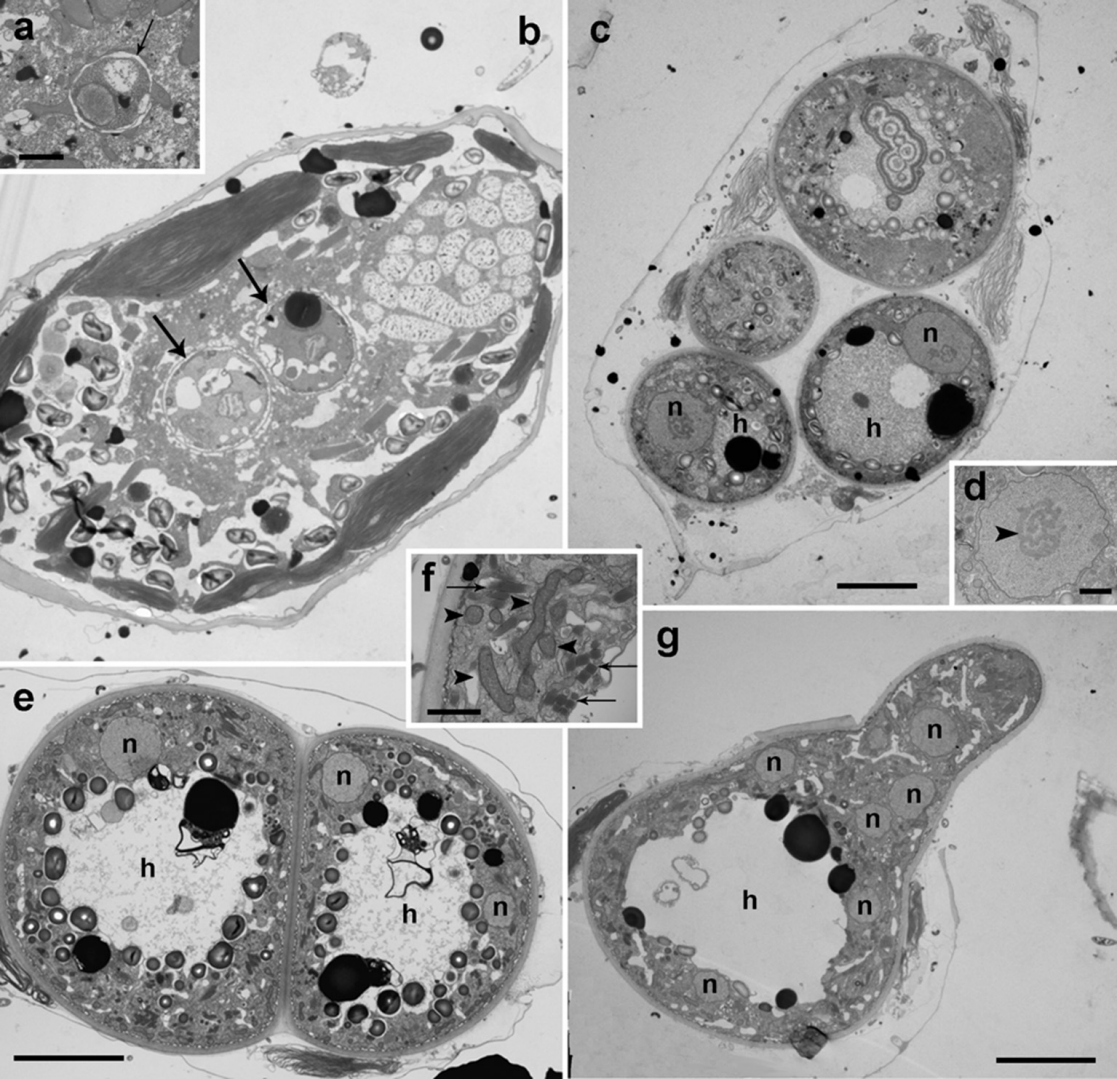
Transmission electron microscopy micrographs showing the life-cycle stages from early infections to early sporocyte of *Dinovorax pyriformis* infecting *Prorocentrum micans*. **(a)** Detail of early infection stage of *D. pyriformis* located inside the parasitophorous vacuole (arrow) at the host cytoplasm. **(b)** Two early trophonts (arrows) infecting a *P. micans* cell. **(c)** Four trophonts after having completely consumed all of the host cytoplasm, showing a large nucleus (n) and central hyaline material (h). **(d)** Detail of the nucleus of the trophont. The nucleolus can be observed (arrowhead). **(e)** Two early sporocytes growing in contact. Two nuclei can be observed in a single sporocyte at this stage (n) and the central hyaline material (h). **(f)** Detail of the cytoplasm with all organelles like mitochondria (arrowheads) and trichocysts (arrows) accumulated at the periphery. **(g)** Early sporocyte forming the germ-tube. Several nuclei (n) can be observed. The central area is occupied by hyaline material (h). All scale bars = 5 μm, except **(a,d,f)** = 1 μm.

**Figure 4:**
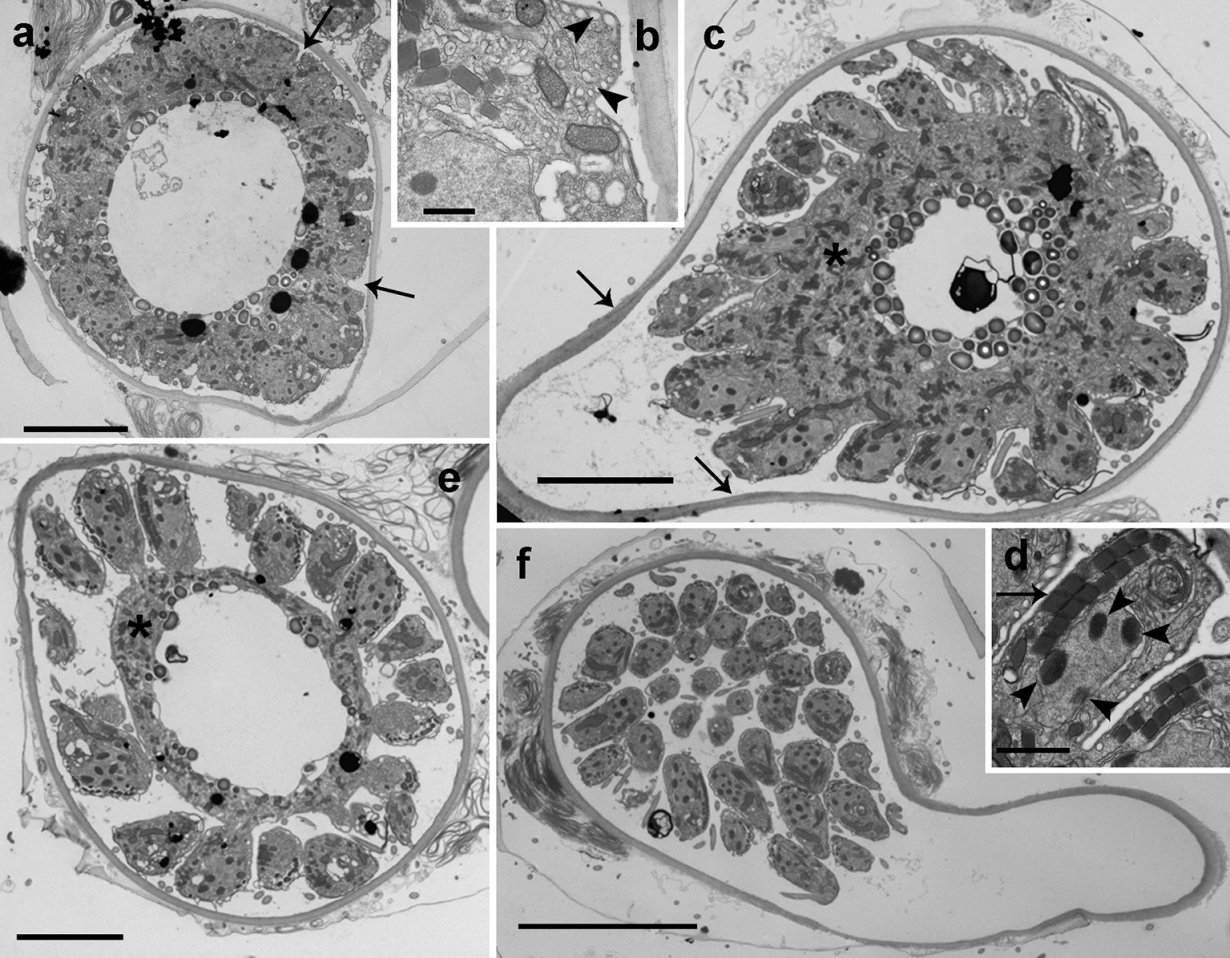
Transmission electron microscopy micrographs showing the life-cycle stages from late sporocyte to sporangium of *Dinovorax pyriformis* infecting *Prorocentrum micans*. **(a)** Late sporocyte with invaginations at the periphery that correspond to initial stage of zoospore cytokinesis (arrows). **(b)** Detail of the invaginations. Alveoli can be observed at a subcortical position (arrowheads). **(c)** Sporocyte with zoospores in formation from the central undifferentiated cytoplasm (asterisk). The arrows indicate the suture among the sporangium and the germ-tube walls. **(d)** Detail of an immature zoospore already showing the characteristic heterochromatin bodies in the nucleus (arrowheads) and two rows of trichocysts (arrow). **(e)** Late sporocyte with zoospores almost formed. The undifferentiated cytoplasm (asterisk) of the sporocyte is gradually consumed. **(f)** Sporangium in longitudinal section containing many mature zoospores detached from the central body. All scale bars = 5 μm, except **(b)** = 500 nm, **(d)** = 1 μm and **(f)** = 10 μm.

**Figure 5:**
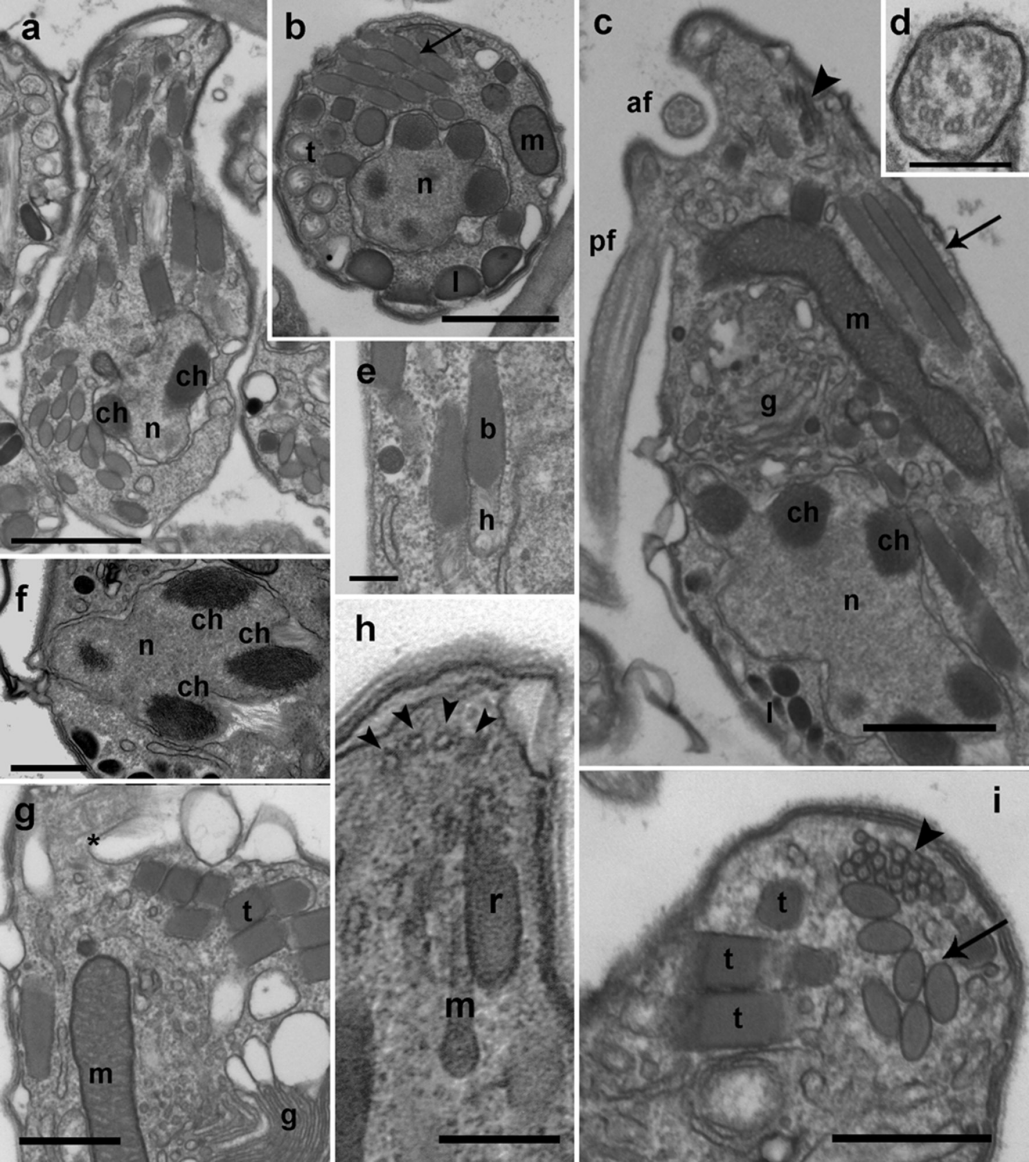
TEM micrographs showing the general features of *Dinovorax pyriformis* zoospores. **(a)** Longitudinal section of a zoospore. The nucleus (n) contains heterochromatin as ovoid bodies (ch). **(b)** Transverse section of a zoospore. The nucleus (n) with heterochromatin occupies a central position, and mitochondrion (m), trichocysts (t), lipid droplets (l), and rhoptries (arrow) are located in the periphery. **(c)** Longitudinal section of a zoospore. The insertion area of the posterior flagellum (pf) and the anterior flagellum (af) are seen. A cluster of micronemes (arrowhead) and rhoptries running longitudinally (arrow) are visible. **(d)** Transverse section of the anterior flagellum axoneme showing a heteromorphic pair of central microtubules. **(e)** Detail of two bipartite trichocysts showing the body (b) and the head (h). **(f)** Detail of the nucleus (n) of a zoospore, showing three heterochromatin ovoid bodies (ch). **(g)** Section of a zoospore showing the Golgi body (g) and the basal body of anterior flagellum (^*^). **(h)** Oblique section of the apical area of a zoospore showing the pseudo-conoid microtubules (arrowheads), a microneme with bulbous end (m) and a rhoptry (r). **(i)** Section of the apical area of a zoospore, showing several trichocysts (t), rhoptries (arrow), and micronemes (arrowhead). Scale bars: **(a,c)** = 1 μm; **(b,f,g,i)** = 500 nm; **(d,e,h)** = 200 nm.

Early stages of infection show one or multiple small spherical trophonts (Figures 3a–c) that grow in size inside a parasitophorous vacuole while degrading the host cytoplasm. The trophonts contain numerous ingestion granules. At this stage, the early trophont contains a large nucleus with visible nucleolus (Figure 3d). After trophont maturation, the large nucleus starts dividing into multiple smaller nuclei without apparent nucleoli (Figures 3e–g). The central area is occupied by hyaline amorphous material (Figure 3e) and all cytoplasmic content is located at the periphery of the sporocyte (Figures 3e–g). Several organelles like mitochondria, trichocysts, and starch grains are already present in its cytoplasm (Figure 3f). Afterwards, the germ-tube with a rounded distal end is formed (Figure 3g). Subsequently, cytokinesis begins and invaginations can be observed at the cell periphery (Figure 4a). All organelles organize in the periphery of the cytoplasm (Figure 4b) and the internal, undifferentiated cytoplasm gradually disappears (Figures 4c,e). The cytoplasm of the sporocyte retracts, leaving the tube empty during successive stages (Figure 4c). At that stage, numerous nuclei are arranged at the cytoplasm periphery (Figure 4c), showing heterochromatin condensed in several ovoid bodies (Figure 4d). When the zoospores are mature, they detach of the central cytoplasm (Figure 4e,f). Finally, the germ-tube opens, and zoospores are released. Observations of multiple infections of different degree of maturation in a single host cell suggest independent infections of the host (not shown). The sigmoid-shaped zoospores (Figure 5a) have a nucleus located posteriorly and occupying a central position in the cell. Nucleus is lobed with several ovoid heterochromatin structures at its periphery (Figures 5a–c,f). The axonema of the anterior flagellum has two dissimilar central microtubules (Figure 5d), and its basal body presents a transverse septum in the transition zone, but lacks of a dense body (Figure 5g). Numerous bipartite trichocysts (ca. 10–20) can be observed, formed by an electron-dense body and a head region (Figure 5e). The body or basal rod is square in cross section (Figure 5g), and the head is circular and formed by twisted filaments (Figure 5b). The Golgi body is located close to the nucleus. The number of cisternae could not be determined with certainty (Figure 5g). A mitochondrion with tubular cristae is located laterally (Figures 5b,g). Numerous starch grains are present and located peripherally (Figures 5b,c). The apical complex (AC) is composed by a pseudo-conoid formed by 4 microtubules in a curvilinear disposition (Figure 5h). Rhoptries and micronemes with bulbous end are also present (Figures 5c,h,i).

### Membranes development in *Dinovorax pyriformis*

**Figure 6:**
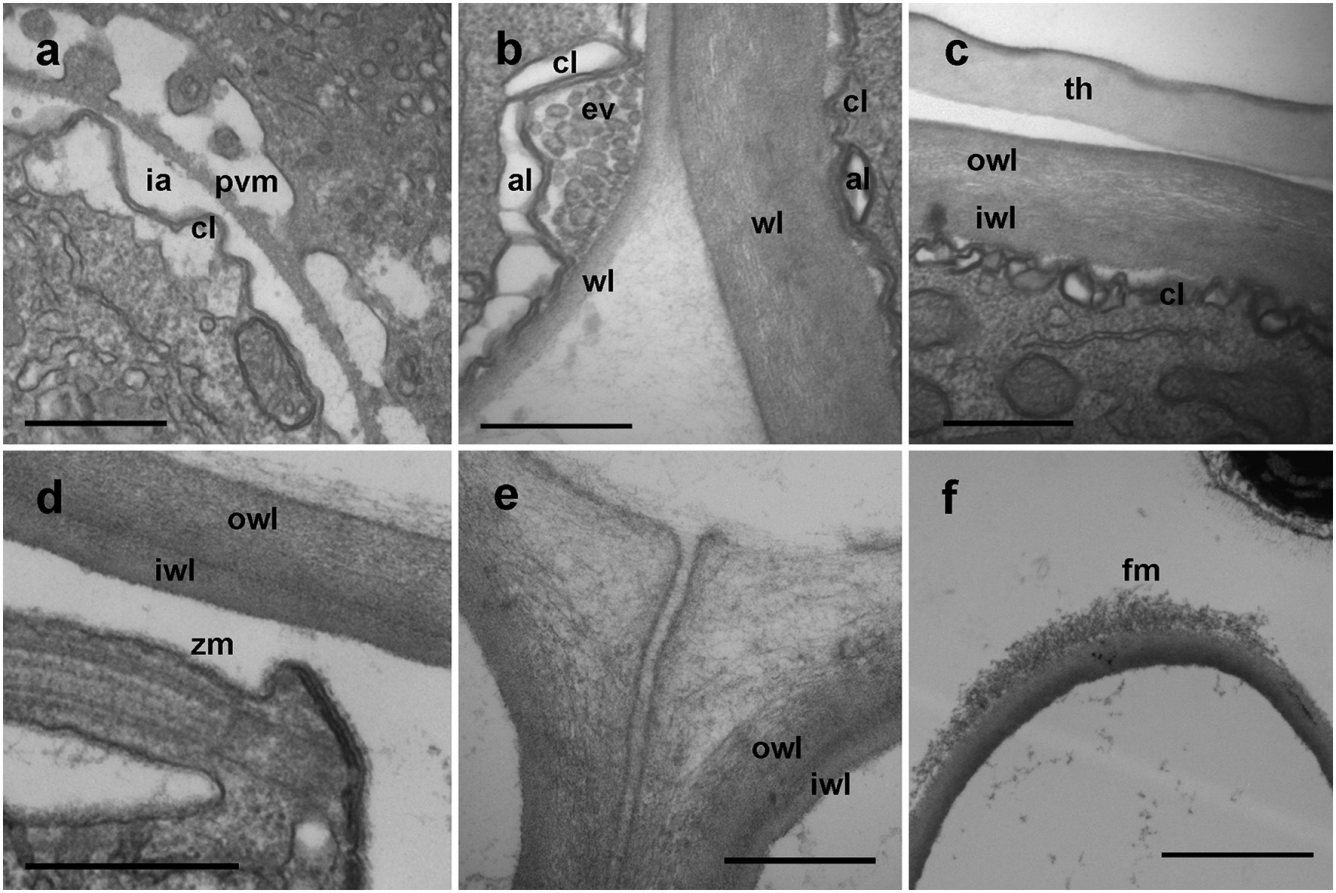
TEM micrographs showing different stages in the development of *Dinovorax pyriformis* membranes. **(a)** Early stage of trophont development showing the parasitophorous vacuole comprising the outer parasitophorous vacuole membrane, an intermediate area and the folded parasite membrane. **(b)** The surface of the parasitoid membrane (cl) is folded, with endo/exocytotic vesicles along the top of the folds. **(c)** Late stage of the trophont, with cyst-wall already formed. **(d)** Cyst-wall structure of a sporocyte. **(e)** Cyst-wall structure of two sporocytes growing in contact. **(f)** Tip of the germ-tube of a sporangium. al, Alveoli; cl, convoluted membrane; ev, endocytotic vesicles; fm, fibrous material; ia, intermediate area; iwl, inner wall layer; owl, outer wall layer; pvm, parasitophorous vacuole membrane; wl, wall layer; th, dinoflagellate theca; zm, zoospore membrane. Scale bars: **(a–e)** = 500 nm; **(f)** = 2 μm.

During early stages of infection, the parasitoid grows inside the parasitophorous vacuole. It is separated from the host cytoplasm by two-layered membranes; an outer membrane known as the parasitophorous vacuole membrane, an intermediate hyaline area, and an inner convoluted membrane representing the plasmatic membrane of the parasite (Figure 6a). Once the early trophont degrades the host cytoplasm, the outer membrane gradually transforms into the cyst wall and the intermediate area is occupied by numerous alveolar vesicles resulting of the digestion of the host cytoplasm (Figure 6b). When the trophont matures, the cyst wall shows two differentiated areas, an internal, electron-dense area, and a thicker, less compact outer area. The cyst wall structure is smooth, with no processes or pores all along the sporangium. The internal convolute layer is maintained, with numerous alveoli present (Figure 6c). Once the germ-tube is formed, the suture among the initial and the newly formed cyst wall can be observed (Figure 4c), with the germ-tube wall originating from the inner side of the sporangium at this suture region. During formation of zoospores, the convoluted membrane becomes the membrane of the individual zoospores (Figure 6d). When multiple infections occur, trophonts can develop individually, with spherical shape, and grow in close contact among them. In that case, the contact area is not rounded but flattened, and it shows a less compact appearance than the outer area of the cyst wall (Figure 6e). At the germ-tube tip, filamentous material is often present (Figure 6f).

### Phylogeny of parviluciferaceae

SSU rDNA sequences obtained for *D. pyriformis* and *Snorkelia* sp. showed a pairwise identity of 91% among them, a 92.1 and 97.1% respectively to *P. prorocentri* sequence, and an 82.4 and 82.3% respectively to the closest *Parvilucifera* sequence (*P. rostrata*).

**Figure 7:**
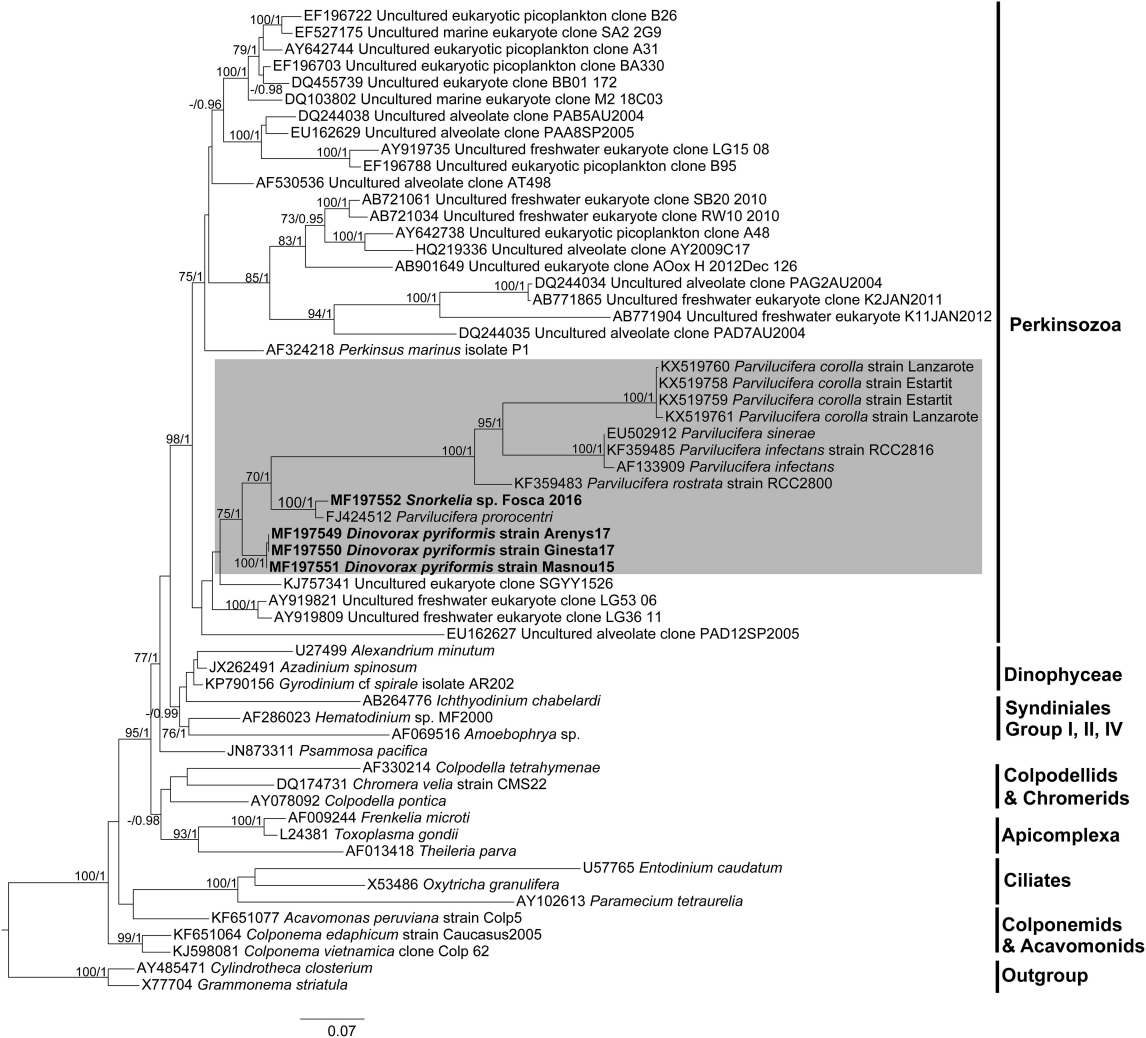
Maximum likelihood phylogenetic tree inferred from the SSU rDNA sequences. Sequences of the diatoms *Grammonema striatula* and *Cylindrotheca closterium* served as outgroups. Sequences obtained in this study are indicated in bold and the shaded area encompasses Parviluciferaceae members. The bootstrap values (BS) and Bayesian posterior probabilities (BPP) are provided at each node (BS/BPP). Only BS and BPP values >70% and >0.95, respectively, are shown.

SSU rDNA phylogeny (Figure 7) has been constructed with the sequences obtained in this study, sequences of other *Parvilucifera* and *Perkinsus* members as well as with environmental sequences available in GenBank, altogether covering the phylogenetic diversity of the Perkinsozoa clade. Representatives of main Alveolata groups have also been included. Colponemids (99% bootstrap/1 Bayesian posterior probability), Acavomonids and ciliates (100%/1) cluster at the base of the Alveolata (100%/1). Then, Myzozoan groups cluster together (95%/1), with Colpodellids, Chromerids and Apicomplexa at the base (−/0.98), and Dinozoa clustering together (77%/1), including *Psammosa pacifica*, Syndiniales representatives, Dinophyceae, and Perkinsozoa. Perkinsozoa form a monophyletic group (98%/1), with two separate clades, one containing *Perkinsus* representatives and a number of environmental sequences (75%/1), and another containing dinoflagellate parasitoids and other environmental sequences. All sequences of *Parvilucifera* representatives, and those obtained in this study cluster together (75%/1). *Dinovorax pyriformis* clusters at the base of the group (100%/1). As a sister clade (70%/1), *P. prorocentri* and *Snorkelia* sp. cluster together (100%/1), while the rest of species (*P. infectans*/*sinerae, P. corolla* and *P. rostrata*) form another highly-supported cluster (100%/1).

The LSU rDNA phylogeny (Figure S1) obtained with available sequences of Perkinsozoa shows that *Perkinsus* and *Parvilucifera* sequences group within the same cluster under moderate support (74%/1), but conforming sister clades. *Dinovorax pyriformis* clusters at the base of the Parviluciferaceae cluster (100%/1), followed by a highly-supported cluster (100%/1) containing *P. infectans*/*sinerae, P. rostrata* and the sequences of *P. corolla* obtained in this study. Unfortunately, the LSU rDNA sequence of *P. prorocentri* and *Snorkelia* sp. are not available for the comparison of its phylogenetic position with the *Dinovorax* sequence.

## Discussion

### Comparison of morphological, ultrastructural and phylogenetic features, and support for the erection of two new genera for *Dinovorax pyriformis* and *Snorkelia* spp.

Both morphological and phylogenetic results obtained in this study support the erection of a new species for *Dinovorax pyriformis*. Based on phylogenetic information, *Snorkelia* sp. represents another new species. However, its culture was lost, and we could only observe that most life-cycle stages were similar to those of *Dinovorax* and that sporangia also formed a germ-tube. Therefore, the scarcity of characters observed for this species does not allow a formal description. All studied features show that *D. pyriformis* and *Snorkelia* sp. have significant distance with the other species of the genus *Parvilucifera*. *Parvilucifera* species form a highly supported phylogenetic clade, including *P. infectans, P. sinerae, P. rostrata*, and *P. corolla*. All of them show high similarity in morphological characters, i.e., sporangium structure, life-cycle development, and main differences refer to the zoospore morphology, and the organization of the zoospores during their maturation. The exception is *P. prorocentri*, which shows higher affinity, both in morphology and phylogeny, for the organisms described in the present work. The main morphological differences observed among *D. pyriformis, Snorkelia* sp., and *P. prorocentri* with the other *Parvilucifera* species include the formation of a germ-tube in the sporangium stage from where the zoospores are released or discharged to the environment. Also, the sporangium cyst wall structure is different, being thicker, smooth, and lacking processes. Moreover, ultrastructural differences are also present (Table 1), such as the presence of bipartite trichocysts, a nucleus with heterochromatin, and the absence of a dense area in the axonemes. Such information supports that *D. pyriformis, Snorkelia* sp. and *P. prorocentri* do not belong to the genus *Parvilucifera*. Actually, Hoppenrath and Leander (2009) pointed out that the phylogenetic distance and morphological differences of *P. prorocentri* with the other *Parvilucifera* species justified its classification within a different genus. However, the scarcity of information available at that moment on Perkinsozoa diversity and ultrastructural features refrained them. Phylogenetic results based on SSU rDNA sequences show that *D. pyriformis, P. prorocentri*, and *Snorkelia* sp. are closely related, but cluster paraphyletically at the base of *Parvilucifera* members (Figure 7). Consequently, we propose to classify them within two different new genera, with *Snorkelia* sp., and *S. prorocentri* (=*P. prorocentri*) belonging to the same genus.

**Table 1:** Comparison of the morphological characters of *Dinovorax pyriformis* with other Perkinsozoa.

	***Dinovorax pyriformis***	***Snorkelia prorocentri*^a^**	***Parvilucifera* spp.^b^^-^^e^**	***Perkinsus* spp.^f^^,^^g^**	***Rastrimonas subtilis*^h^**
Alveoli	+	+	+	+	+
Mitochondrion with tubular cristae	+	+	+	+	−
Refractile body in zoospore	−	+	+	+	−
germ-tube	+	+	−	+	−
Presence of processes in the sporangium	−	−	+	−	No sporangium
Bipartite trichocysts	+	+	−	−	−
Heterochromatin formation	Early sporocyte	Late sporocyte	−	−	?
Heterochromatin in zoospores nucleus	+	+	−	−	+
Heterochromatin distribution	Ovoid bodies	Peripheral	−	−	Irregular bodies
Zoospores shape	Sigmoid	Reniform	Reniform/Elongated	Reniform	Elongated
Two dissimilar flagella	+	+	+	−	+
Short posterior flagellum	−	+	+	−	−
Heteromorphic pair of central					
microtubules in anterior axoneme	+	+	+	+	?
Dense globule in the basal body	−	−	+/?	+	−
Dense area in the axoneme	−	−	+	−/+	−
Micronemes	+	+	+	+	+
Rhoptries	+	+	+	+	?
Reduced pseudoconoid (n° of microtubules)	+ (4)	+ (4–6)	+ (4–5)	− (20–39)	+ (4–5)
Conoid-associated micronemes	?	+	+	+	+

a *Leander and Hoppenrath (2008)*.

b *Norén et al. (1999)*.

c *Garcés and Hoppenrath (2010)*.

d *Lepelletier et al. (2014)*.

e *Reñé et al. (2017)*.

f *Azevedo (1989)*.

g *Casas et al. (2004)*.

h *Brugerolle (2002)*.

Nevertheless, some remarkable differences are found among *S. prorocentri* and *D. pyriformis*. Zoospores of *D. pyriformis* are sigmoid and have both posterior and anterior flagella similar in length, while *S. prorocentri* zoospores are reniform and the posterior flagellum is shorter. At ultrastructural level, *Dinovorax* and *Snorkelia* show a different organization of heterochromatin in the zoospores nucleus. It has a peripheral distribution in *S. prorocentri*, while it is present as ovoid structures scattered along the nucleus periphery in *D. pyriformis*. Furthermore, the early sporocytes of *S. prorocentri* contain nuclei without heterochromatin, while it is already present at early stages in *Dinovorax*. Other additional differences refer to the presence of a refractile body in the zoospores, or the ultrastructural organization of zoospores organelles. We commonly observed multiple infections of *D. pyriformis* on *Dinophysis sacculus* (6–8), but only double infections of *D. pyriformis* on *P. micans*. Only double infections were observed for *Snorkelia* sp. when infecting *L. fissa*, as also reported for *S. prorocentri* when infecting *Prorocentrum fukuyoi* (Leander and Hoppenrath, 2008). This suggests that this difference may not be intrinsic to the causative parasitoid, but to the infected host species. The number of zoospores produced significantly differs among species. While *Dinovorax* produces less than a hundred new zoospores, other species like *P. sinerae, P. infectans*, or *P. corolla* release several hundreds of zoospores for sporangia of similar biovolume (Norén et al., 1999; Garcés et al., 2013; Reñé et al., 2017).

Finally, this group of organisms, including *Parvilucifera* representatives, shares many characters in common, including that all of them are parasitoids of dinoflagellates, the overall development and characteristics of their life-cycle stages, and their infection strategy. Thus, we propose to include all of them into the family Parviluciferaceae. The knowledge on Perkinsozoa diversity is so scarce that new species and probably, new genera will be further included within this family as more studies are conducted. The other species of Perkinsozoa described to date are *R. subtilis* and those belonging to *Perkinsus*. Phylogenetic information of *R. subtilis* is not available, and *Perkinsus* representatives does not cluster within Parviluciferaceae. Nonetheless, the freshwater species *R. subtilis* shows a life-cycle similar to Parviluciferaceae, but zoospores are formed directly within the host cytoplasm, not being surrounded by a sporangium (Brugerolle, 2002). *Perkinsus* species present a more complex intracellular life-cycle, including several intermediate stages like hypnospores and prezoosporangia, and develop in molluscan tissues (Sunila et al., 2001; Casas et al., 2004). Further morphological differences with Parviluciferaceae are listed in Table 1.

### Evolutionary framework of morphological characters among parasitic groups of alveolates

**Figure 8:**
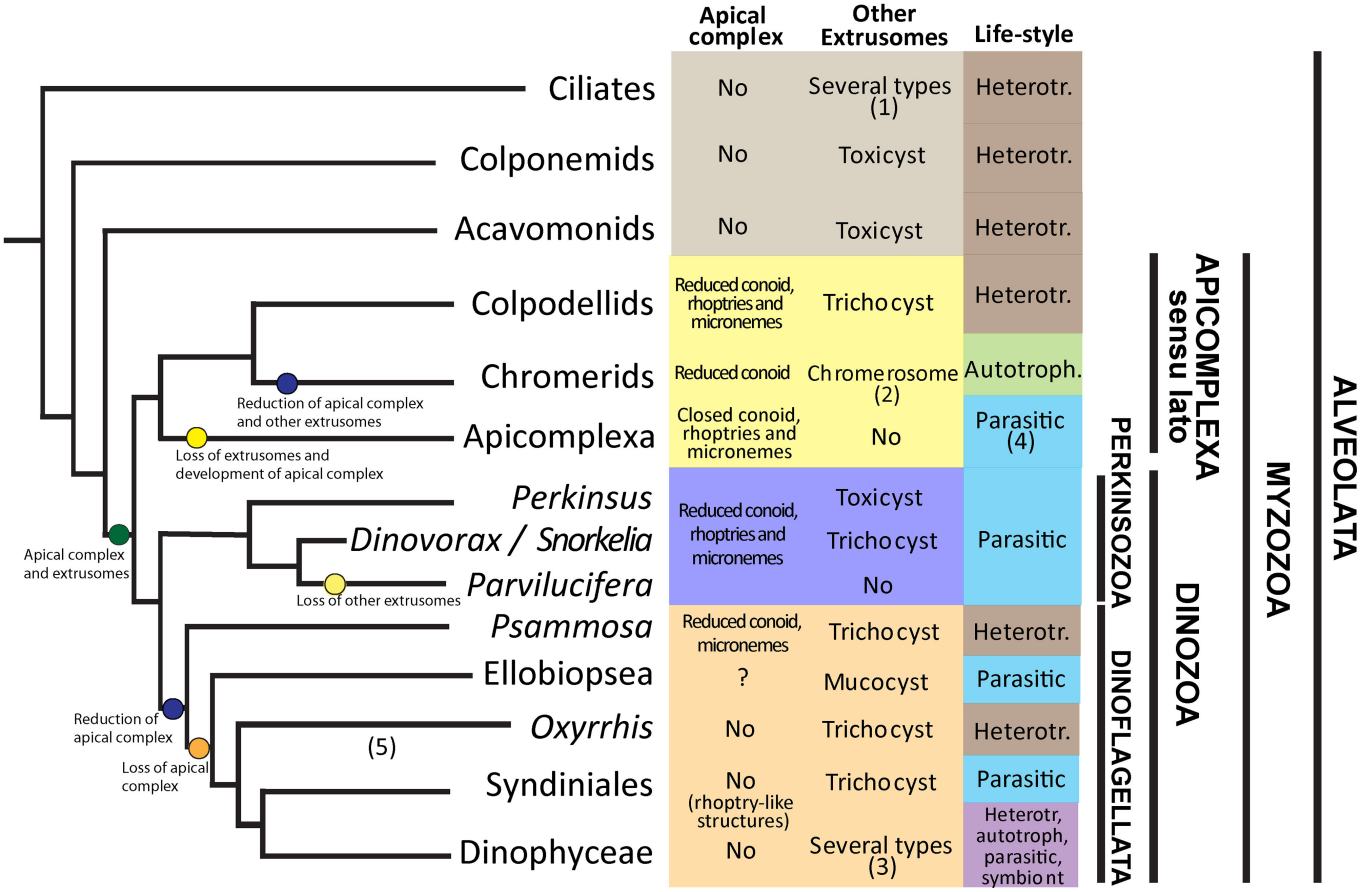
Framework of extrusomes, apical complex and life-style evolution among Alveolates. (1) Trichocysts, mucocysts, toxicysts among others. (2) Chromerosome shows similitude with toxicysts. (3) Trichocysts, mucocysts, taeniocysts, nematocysts. (4) Parasitic, but present the apicoplast, a plastid relic. (5) The phylogenetic position is based on Bachvaroff et al. (2014) and Janouškovec et al. (2017). Heterotr. = heterotroph.

Alveolata is a superphylum comprising many diverging groups of protists, including ciliates, Apicomplexa or Dinophyceae (Figure 8). Ciliates occupy the basal position, together with the predatory Colponemids and Acavomonids. The remaining organisms cluster in the Myzozoa clade. Two sister branches of Myzozoa are currently known, the Apicomplexa *sensu lato* clade and the Dinozoa. Within Dinozoa, Perkinsozoa occupy a basal position suggesting their features represent ancestral characters for all Dinozoa and Myzozoa representatives. Among Alveolata groups, Dinophyceae encompasses parasitic organisms, and other groups like Apicomplexa, Syndiniales, and Perkinsozoa are exclusively parasitic. Although numerous environmental sequences are available for Perkinsozoa, only the morphology, life-cycle and ultrastructure of *Perkinsus* species, *R. subtilis*, and members of Parviluciferaceae are known, all of them having a parasitic life-style. Parviluciferaceae and *Perkinsus* cluster in two sister clades that have evolved independently from a common ancestor. Within Parviluciferaceae, *Dinovorax* and *Snorkelia* occupy a basal position and maintain ancestral characters compared to *Parvilucifera* members. While parasitic behavior has evolved independently in different groups of alveolates, some characters are shared among them.

A common character of Alveolates is the presence of extrusomes. Such extrusomes have been related to a variety of functions, including defense, attachment or immobilization of prey (Rosati and Modeo, 2003). Ciliates are predators possessing numerous extrusomes of several types, i.e., trichocysts, toxicysts, and mucocysts (Rosati and Modeo, 2003). The predatory behavior and the presence of extrusomes is maintained in other basal phyla like Colponemids and Acavomonids (Tikhonenkov et al., 2014). The common ancestor of Myzozoans likely possessed plastids (Oborník and Lukes, 2015), extrusomes and an apical complex involved in the uptake of nutrients from a prey (Leander and Keeling, 2003). Evidences for the presence of plastids as an ancestral character emerge because some groups like Chromerids or Dinophyceae are plastidial, and others show or likely possess a plastid-relic, like Apicomplexa, *Oxyrrhis* or *Perkinsus* (Oborník et al., 2009). The apical complex (AC) is composed of several structures including micronemes, a number of microtubules namely conoid, and extrusive rhoptries, that are related to host or prey penetration and invasion (Gubbels and Duaraisingh, 2012), and is present in all basal groups of Myzozoa. However, its structure varies from a “reduced” AC, consisting of an open- or pseudoconoid and several vesicular organelles, to an “evolved” AC with a closed conoid, including some other intermediate forms. Some evolutionary trends are observed in these characters for the different groups of Myzozoa. Representatives of Apicomplexa *sensu lato* like Colpodellids maintain most features, including an intermediate AC with rhoptries, but lost the plastid. Chromerids possess a plastid, and an AC with rhoptries but they do not possess any kind of extrusomes. However, they present a so-called “chromerosome,” showing structural resemblance with extrusomes or trichocysts (Oborník et al., 2011). Finally, Apicomplexa possess a well-developed apical complex with a closed conoid and extrusive rhoptries, a plastidial relic, called apicoplast, confirming the hypothesis of a pigmented common ancestor of Myzozoa (Oborník et al., 2009), but have lost other extrusomes.

Among Dinozoa, Parviluciferaceae, *Rastrimonas* and *Psammosa* present a reduced AC (Norén et al., 1999; Brugerolle, 2002; Okamoto and Keeling, 2014a), while the AC of *Perkinsus* is more developed (Sunila et al., 2001), resembling that of Colpodellids (Leander and Keeling, 2003). By contrast, Syndiniales and Dinophyceae have completely lost these structures (Leander and Keeling, 2003), although some Syndiniales like *Amoebophrya* possess electron-dense bodies that show functional similarities to rhoptries (Miller et al., 2012). Okamoto and Keeling (2014b) suggested that the AC and the peduncle present in some dinoflagellates could be homologous. Conversely, extrusomes not related with the AC are present in all phyla of Dinozoa, including some Perkinsozoa, *Psammosa* (Okamoto et al., 2012), Ellobiopsidae (Gómez and Horiguchi, 2014), Syndiniales (Skovgaard et al., 2005), and Dinophyceae (Taylor, 1980). Dinophyceae present the most diverse life-style behaviors, including heterotrophy, autotrophy, mixotrophy, parasitism and symbiosis. Some dinoflagellates, similarly to ciliates, present different kinds of extrusomes, i.e., *Polykrikos* representatives (Hoppenrath et al., 2010). Perkinsozoa, that occupy a basal position in Dinozoa, evolved into a parasitic life-style. Interestingly, extrusomes (trichocysts or toxicysts) and a reduced AC with extrusive rhoptries are present in *Perkinsus, Dinovorax*, and *Snorkelia* (Casas et al., 2004; Leander and Hoppenrath, 2008). However, *Parvilucifera* spp. and *Rastrimonas* present an intermediate AC with extrusive rhoptries, but other extrusomes have been lost in *Parvilucifera* spp. (Lepelletier et al., 2014; Reñé et al., 2017) or not observed in *Rastrimonas* (Brugerolle, 2002). Based on phylogenetic analyses, *Parvilucifera* occupy a terminal position in Parviluciferaceae, suggesting that they are more evolved organisms.

Additionally, *Dinovorax* and *Snorkelia* develop a germ-tube in the sporangium. This feature is shared with *Perkinsus* spp. (Sunila et al., 2001), but it has been lost in *Parvilucifera* spp., which zoospores are released via opercula (Norén et al., 1999; Lepelletier et al., 2014; Alacid et al., 2015; Reñé et al., 2017). The germ-tube and the sporangium present a thick cyst-wall, as observed for *Perkinsus* spp. (Sunila et al., 2001; Casas et al., 2004) in contrast to the *Parvilucifera* sporangia, which show a thinner wall with numerous processes. By contrast, *Rastrimonas* does not grow inside a parasitophorous vacuole and does not form a sporangium during the formation of zoospores. A similar thick cell-wall is present in the ectoparasite *Ellobiopsis* (Gómez and Horiguchi, 2014), the closest group to Perkinsozoa, but it is absent in other Dinozoa groups. Thus, as previously suggested by Hoppenrath and Leander (2009), the presence of a germ-tube in the sporangium can also be considered as an ancestral character of a common ancestor of Dinozoa.

A further comparison could be performed for flagella characteristics. Zoospores of *Dinovorax* present two flagella similar in length, while the other Parviluciferaceae have a reduced posterior flagellum. In any case, the flagella are heteromorphic, with a haired transverse flagellum. Nevertheless, the flagella characteristics do not seem to show an evolutionary trend among Dinozoa groups. The genera *Psammosa* and *Oxyrrhis*, which show an intermediate phylogenetic position between Perkinsozoa and Dinophyceae, have a short anterior and a long posterior flagellum, no undulation of either one, and both flagella haired (Okamoto et al., 2012). Only the anterior transverse flagellum is haired for members of Syndiniales like *Amoebophrya* (Miller et al., 2012), and *Hematodinium* (Appleton and Vickerman, 1998). *Syndinium turbo* shows microspores with a longer and haired anterior flagellum, and macrospores with both flagella naked and similar in length (Skovgaard et al., 2005). Finally, Dinophyceae possess a characteristic coiling transverse flagellum encircling the cell (Taylor, 1980). The anterior flagellum encircling the cell body in *Dinovorax* could also be considered as a precursor of the coiling transverse flagellum of Dinozoa.

There is an evident variability in nuclei organization of Perkinsozoa. Zoospores of *Parvilucifera* and *Perkinsus* species have a conventional nucleus with fibrous material in its periphery (Sunila et al., 2001; Lepelletier et al., 2014; Reñé et al., 2017). *Snorkelia prorocentri* shows a nucleus with heterochromatin along its periphery (Leander and Hoppenrath, 2008). However, *Dinovorax* zoospores show a particular characteristic, with heterochromatin packed in ovoid bodies scattered along the nucleus periphery. *Rastrimonas* nucleus shows intermediate characteristics among *Dinovorax* and *Snorkelia* (Brugerolle, 2002).

This evolutionary framework suggests that from a photosynthetic ancestor possessing trichocysts and an apical complex, some groups as Apicomplexa and Perkinsozoa have specialized into parasitic life-styles, losing their plastids, developing the apical complex structures devoted to the host penetration while losing other extrusomes. By contrast, although some groups of Syndiniales are exclusively parasitic, Dinoflagellata representatives have diversified their life-styles, including the parasitic one. Accordingly, Dinophyceae, which encompass many different life-styles including autotrophy, heterotrophy, mixotrophy, symbioses and parasitism, have lost or transformed the apical complex structures related to attachment or penetration to prey, while maintained and developed their other extrusomes.

In summary, a new species of parasitoid has been described in this study, leading to a reclassification of *Parvilucifera* representatives and the erection of two new genera. Based on their phylogenetic position and the morphological features observed, the hypothetical evolutionary trends occurred within this group have been inferred.

## Author contributions

AR, EA, and EG conceived and designed the study and performed the samplings; AR and EA performed the microscopy preparations and observations; AR, EA, and IF performed molecular analyses; All authors analyzed and interpreted the data, wrote the paper and reviewed the manuscript.

### Conflict of interest statement

The authors declare that the research was conducted in the absence of any commercial or financial relationships that could be construed as a potential conflict of interest. The reviewer LG and handling Editor declared their shared affiliation, and the handling Editor states that the process nevertheless met the standards of a fair and objective review.

## Supplementary material

Figure S1 Maximum likelihood phylogenetic tree inferred from the LSU rDNA phylogeny. Sequences of ciliates served as the outgroup. Sequence of *Dinovorax pyriformis* obtained in this study is indicated in bold and the shaded area encompasses Parviluciferaceae members. The bootstrap values (BS) and Bayesian posterior probabilities (BPP) are provided at each node (BS/BPP). Only BS and BPP values >70% and >0.95, respectively, are shown.

Video S1 Two days time-lapse of infection development of *Dinovorax pyriformis* on the dinoflagellate *Prorocentrum micans*.

Video S2 Zoospores of *Dinovorax pyriformis* being released through the germ-tube of the sporangium.

Table S1 Location and date of isolation of *Dinovorax pyriformis, Snorkelia* sp. and *Parvilucifera corolla* strains, with a detail of strains used for morphological and ultrastructural observations presented, and the correspondence of genetic sequences obtained.
