# Supplementary figures and images for: Evolutionary Trends of Perkinsozoa (Alveolata) Characters Based on Observations of Two New Genera of Parasitoids of dinoflagellates, Dinovorax gen. nov. and Snorkelia gen. nov
Source: Front Microbiol. 2017 Aug 24;8:1594. doi: 10.3389/fmicb.2017.01594 (PMC5609580; doi:10.3389/fmicb.2017.01594)

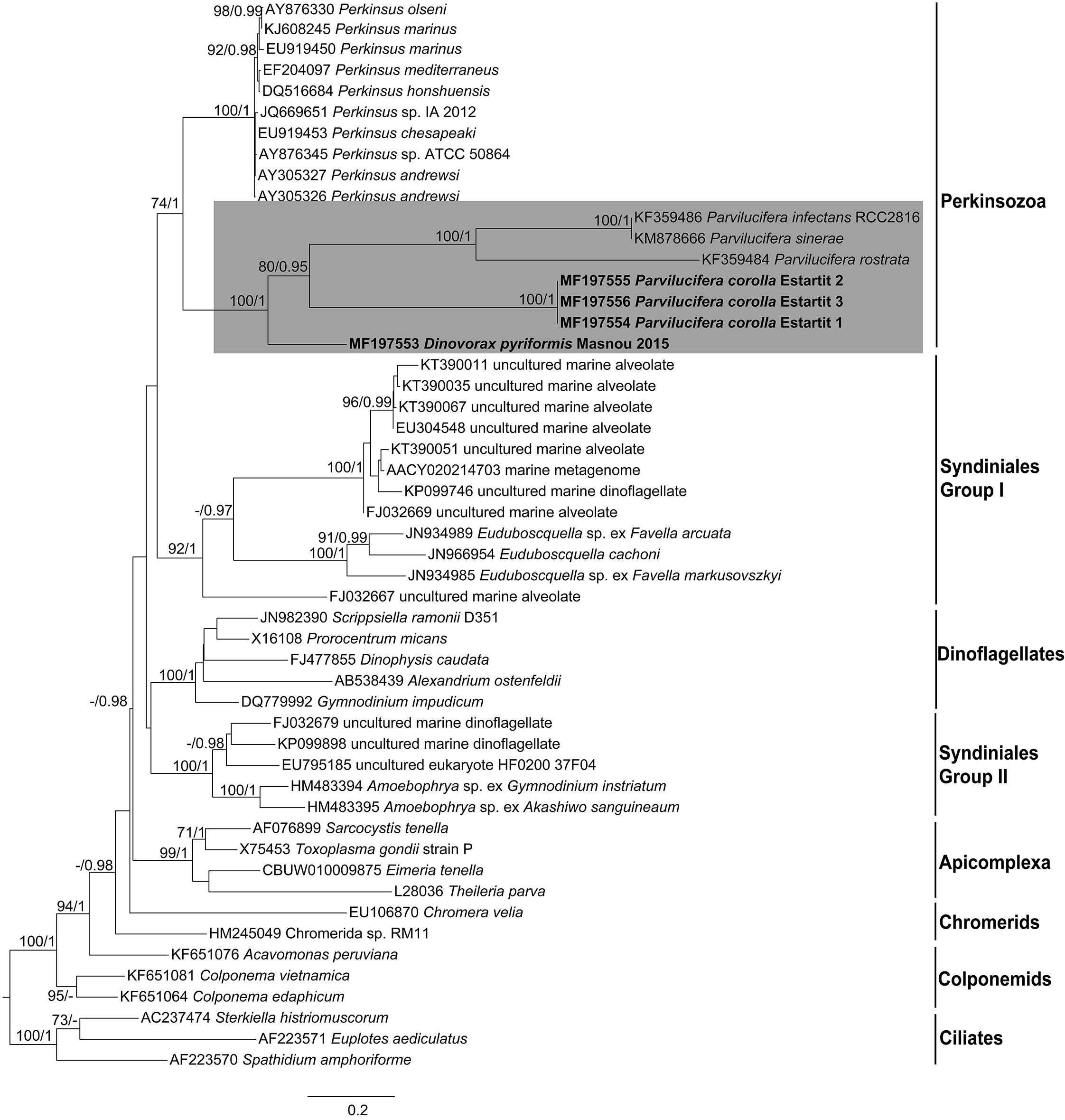

Supplement: Figure S1 — Maximum likelihood phylogenetic tree inferred from the LSU rDNA phylogeny. Sequences of ciliates served as the outgroup. Sequence of Dinovorax pyriformis obtained in this study is indicated in bold and the shaded area encompasses Parviluciferaceae members. The bootstrap values (BS) and Bayesian posterior probabilities (BPP) are provided at each node (BS/BPP). Only BS and BPP values >70% and >0.95, respectively, are shown. [file Image1.TIF]
