# Supplementary material for: Evolutionary Trends of Perkinsozoa (Alveolata) Characters Based on Observations of Two New Genera of Parasitoids of dinoflagellates, Dinovorax gen. nov. and Snorkelia gen. nov
Source: Front Microbiol. 2017 Aug 24;8:1594. doi: 10.3389/fmicb.2017.01594 (PMC5609580; doi:10.3389/fmicb.2017.01594)
Supplement: Table S1 — Location and date of isolation of Dinovorax pyriformis, Snorkelia sp. and Parvilucifera corolla strains, with a detail of strains used for morphological and ultrastructural observations presented, and the correspondence of genetic sequences obtained. [file Table1.DOCX]

**Table S1: Location and date of isolation of *Dinovorax pyriformis*, *Snorkelia* sp. and *Parvilucifera corolla* strains, with a detail of strains used for morphological and ultrastructural observations presented, and the correspondence of genetic sequences obtained.**

| Strain | Location | Date of isolation | Host in nature | LM | SEM | TEM | SSU rDNA sequence | LSU rDNA sequence |
| --- | --- | --- | --- | --- | --- | --- | --- | --- |
| *Dinovorax pyriformis* Masnou 2015 | El Masnou Harbor | March 2015 | *Dinophysis sacculus* | x | x | x | MF197551 | MF197553 |
| *Dinovorax pyriformis* Masnou 2016 | El Masnou Harbor | January 2016 | *D. sacculus* | x |  |  |  |  |
| *Dinovorax pyriformis* Arenys 2017 | Arenys de Mar Harbor | January 2017 | *D. sacculus* |  |  |  | MF197549 |  |
| *Dinovorax pyriformis* Ginesta 2017 | Ginesta Harbor | January 2017 | *D. sacculus* |  |  |  | MF197550 |  |
| *Snorkelia* sp. Fosca 2016 | La Fosca beach | August 2016 | *Levanderina fissa* | x |  |  | MF197552 |  |
| *Parvilucifera corolla* Estartit | Fra Ramon salt marsh | May 2015 | *Durinskia baltica* |  |  |  |  | MF197554 MF197555 MF197556 |
